# MRI Abnormalities of the Brain After Complex Febrile Seizures in Children

**DOI:** 10.7759/cureus.33084

**Published:** 2022-12-29

**Authors:** Rakesh Das, Banashree Swain, Chinmaya K Sahoo, Pradeep K Jena, Bikash R Sahu, Narendra N Soren, Shantibhusan Das, Nirmal K Mohakud

**Affiliations:** 1 Pediatrics, Sriram Chandra Bhanj (SCB) Medical College, Cuttack, IND; 2 Biochemistry, Sriram Chandra Bhanj (SCB) Medical College, Cuttack, IND; 3 Pediatrics, Saheed Laxman Nayak (SLN) Medical College and Hospital, Koraput, IND; 4 Zoology, Centurion University of Technology and Management, Bhubaneswar, IND; 5 Pediatric Medicine, Kalinga Institute of Medical Sciences, Bhubaneswar, IND

**Keywords:** csf, acute disseminated encephalomyelitis, status epilepticus, gtcs, cortical hyperintensity, mri, febrile seizures

## Abstract

Background

Though simple febrile seizures do not cause significant and lasting neurological deficits, complex febrile seizures (CFS) can result in neurologic sequelae. Because CFS causes cortical focal injuries and other brain lesions, it needs to be evaluated.

Objective

The objective of this study was to evaluate the MRI brain changes in CFS, the incidence of seizures in children aged six months to five years, and their severity in relation to MRI findings of the brain.

Methods

In this observational study, 36 children aged six months to five years, having fever with seizure, and fulfilling the criteria of CFS were enrolled within 48 hours of the episode. Detailed clinical and neurological examinations were performed with MRI scans to find out the probable CNS lesions of CFS. Two radiologists separately evaluated all MRI brains.

Results

MRI lesions of the brain were found in 11 (30.56%) CFS patients. Generalized tonic-clonic seizures (GTCS) (n=31, 86.11%) were the most common type of seizure among CFS cases. Cortical focal hyperintensity (CFH) (42.1%) was the most common MRI presentation. Positive MRI findings were significantly associated with focal convulsions (n=5, 100%) as compared to GTCS cases (n=6, 19%) (p=0.001).

Conclusions

CFH is the most common abnormality in the MRI brain among CFS cases. CFS patients with focal seizures or prolonged seizures in 24 hours have higher abnormal neuroimaging findings. MRI should be considered a preferred investigatory tool for detecting CNS pathology in CFS cases. Follow-up studies are needed to determine the long-term outcomes of CNS lesions in children with CFS.

## Introduction

A febrile seizure is a type of clinical manifestation characterized by a burst of uncontrolled electrical activity in brain cells. It is characterized by fever, without an infection of the central nervous system, or a history of afebrile seizures or neurologic dysfunction. Febrile seizures in the pediatric population range from 2-5% of children aged six months to five years [1]. Febrile seizures (FS) are categorized as simple febrile seizure (SFS), complex febrile seizure (CFS), and febrile status epilepticus (FSE). An SFS is typically generalized, occurs once every 24 hours, and lasts no longer than 15 minutes [2]. Among children with febrile seizures, about 70% to 75% demonstrate simple febrile seizures [3]. A febrile seizure is considered complex if it is focal or localized to a specific part of the body, lasts 15 to 30 minutes, or involves a recurrence of seizures within a 24-hour period [4]. As per a report in Iran, between 20 and 25 percent of febrile seizures are complex [3].

Simple febrile seizures typically do not result in significant or long-lasting brain damage [5]. In a few case reports where febrile seizures resulted in permanent or severe brain injury, underlying encephalitis or encephalopathy was likely the source of neurologic disability [6,7]. Rarely, febrile seizures may result in neurologic sequelae unless associated with extremely high fever, unusual severity, and duration. With complex febrile seizures, the risk of long-term neurologic problems is also low; however, the neuroimaging findings need to be evaluated for possible underlying factors.

For accurate diagnosis of FS, several medical techniques and protocols are available. Magnetic resonance imaging (MRI) of the brain employs a strong magnetic field, radio waves, and a computer to generate structural details of the brain that are far more useful and provide more adequate information than other imaging systems. In complex febrile seizures, the standard textbook regimen suggested, as far as possible, a single first-line antiepileptic treatment as the preferred mode. It is very often observed that patients with complex febrile seizures not only require the first line but also need a second line of antiepileptic drugs (AED) to control seizures [8]. It is also observed that the majority of children with CFS require multiple AED to control seizures, even though they sometimes need ICU care to control their febrile status [8]. Hence, there is a need to diagnose the complex febrile seizure early for optimal treatment. MRI is superior (compared to which other techniques) due to anatomic resolution and the characterization of pathologic processes, versatility, and lack of radiation [9]. The need of the current study is to delineate the MRI findings of complex febrile seizures in children for the early diagnosis of CFS so that it can be a useful tool to decide the appropriate treatment. This will help decrease morbidity and mortality among children with CFS.

## Materials and methods

This hospital-based observational study was conducted from December 2019 to November 2021 in the Sardar Vallabh Patel Post Graduate Institute of Pediatrics and Department of Radiodiagnosis, SCB Medical College, Cuttack, Odisha. A total of 36 children aged six months to five years who presented with fever with seizure and fulfilled the criteria of CFS were enrolled in the study. CSF was defined as patients with a fever > 38°C, two episodes of seizures lasting between 15 and 30 minutes, and multiple seizures within a 24-hour period in previously neurologically intact patients or with focal seizure characteristics [2]. Before the study, institutional ethical committee clearance (IEC Appln. No-594) and informed consent from parents were obtained.

Children with seizures occurring for >30 minutes, known cases of epilepsy, signs of central nervous system infection or inflammation, with obvious metabolic abnormality and clear neurologic abnormality leading to seizures (head trauma, brain tumor, or intracranial bleed), children with developmental delay, cerebral palsy, and mental retardation were excluded. Detailed clinical and neurological examinations were performed and further study was conducted with MRI scans to find the probable CNS lesions of CFS. Other investigations were performed as per the clinical history and neurological findings to rule out any CNS infection, metabolic causes, toxin-induced, or any other appropriate causes. Data were recorded in pre-designed proforma for the study. Based on the radiological features, and clinical presentation, a final diagnosis was made. Treatment in the form of supportive measures and specific therapeutic agents administered. Routine investigations like complete blood count, blood urea, and serum creatinine. sodium, potassium, calcium, cerebrospinal fluid (CSF) analysis, and routine, and microscopy examination of urine were done.

MRI scan of the brain (plain/contrast): As soon as feasible, within 72 hours of the initial FS, an MRI examination was conducted on a 1.5 Tesla magnet, but the time period was not more than one week after the seizure. This window period was chosen to precisely study MRI alterations caused by an abrupt seizure. One week appears to be the maximum window for detecting these anomalies, according to experimental results on animal models [10]. Following the appropriate hospital and departmental rules and protocols for sedation of pediatric patients, or children in our study, were evaluated, sedated with either IV midazolam or IV ketamine, monitored, and finally discharged from the department of radiology. The MRI was done in the following order: initially, T1-weighted sagittal scout localizing images were taken, and then T2-weighted proton-density fast spin-echo axial and coronal slices of 5 mm thickness were taken without any gap. Fluid-attenuated inversion recovery (FLAIR) axial images with a slice thickness of 4 mm at a gap of 1 mm and T2-weighted (high resolution) fast spin-echo coronal images by continuous 3 mm slices of the temporal lobes were taken thereafter. A contiguous 1.5 mm slices of the whole brain using volumetric T1-weighted gradient-echo coronal images were evaluated. By using ultrahigh-resolution T2-weighted fast multiplanar inversion-recovery axial images 4 mm thick slices of 1 mm gap were taken from the frontal and parietal lobes. Two radiologists separately evaluated all MRI brains.

Statistical analysis

The data were entered into Microsoft Excel 2010 (Microsoft Corporation, Redmond, WA) and statistical analysis was done using SPSS software version 23.0 (IBM Corp., Redmond, WA). Proportions were taken in cases of categorical variables, and Pearson's chi-square test was used to compare the association of seizure types among positive MRI findings among CFS cases. A p-value <0.05 was considered statistically significant. For continuous variables, mean and standard deviations were calculated.

## Results

Out of 36 children with CFS, there were 25 males (69.44%) (M: F; 2.3:1). The mean age of presentation was 1.93 ± 0.85 years. MRI lesions of the brain were noted in 11 (30.56%) patients. GTCS was the most common type of seizure (n=31, 86.11%) among CFS cases. All cases had normal CSF findings except one. Cortical focal hyperintensity (CFH) (42.1%) was the most common MRI presentation. The number of multiple seizures was 22 (61.11%), and the number of single episodes of seizure was 14 (38.89%) (Table 1).

**Table 1:** Demographic and pathological features in CFS patients during the investigation GTCS, generalized tonic-clonic seizure; CFH, cortical focal hyperintensity; SCFH, sub-cortical focal hyperintensity; AWH, abnormal white matter signal; FCD, focal cortical dysplasia

Variables during investigation	Numbers of patients	Percentage (%)
MRI findings		
Positive cases	11	30.56
Negative cases	25	69.44
Gender		
Male	25	69.44
Female	11	30.56
Age distribution		
0-2.5 yrs	18	50.00
2.5-5yrs	18	50.00
Pattern of seizures		
Focal	5	13.89
GTCS	31	86.11
Types of seizures		
Focal multiple	4	11.11
Focal single	1	2.78
GTCS multiple	18	50.00
GTCS single	13	36.11
Seizure multiplicity		
Multiple	22	61.11
Single	14	38.89
Postictal deficit		
No	30	83.33
Yes	6	16.67
Distribution of CSF cells		
Abnormal	1	2.78
Normal	35	98.72
Presence of MRI features		
CFH	8	42.10
SCFH	5	26.31
AWH	3	15.78
FCD	3	15.78
Specific treatment was there for cases		
Yes	7	63.63
No	4	36.37

In this study, the most common MRI abnormality was cortical focal hyperintensity, which constitutes eight (42.10%) cases; however, there were five (26.31%) subcortical focal hyperintensity cases. The incidence of subcortical white matter abnormalities in this study is 3 (15.71%). Further, the frequency of focal cortical dysplasia in this study is 3 (15.71%). The overall treatable cases (medically or surgically) in our study group were 29 (80.56%) out of a total of 36 patients. In these subjects, the treatable cases as per positive MRI findings were four (36.36%) and untreatable cases were 7 (63.63%). We found the mean duration of hospitalization in subjects with MRI findings to be 12.6+2.8 days and the mean duration of seizure in patients with positive MRI to be 20+ 5.3 min. The longer duration of stay in patients with a positive MRI is due to the longer course of illness, multiplicity of seizures, and time to stabilize the patients before taking neuroimaging (Table 1).

Among the 36 patients, 25 (69.44%) patients had normal neuroimaging. Among cases with abnormal neuroimaging findings, two (5.56%) had acute infarction of the right MCA territory, one (2.78%) had subacute infarction of the right frontal lobe, one (2.78%) had MRI features suggestive of tuberous sclerosis, one (2.78%) had early leukodystrophy changes, one (2.78%) patient had frontotemporal atrophy. However, one (2.78%) patient had acute disseminated encephalomyelitis (ADEM) (Table 2).

**Table 2:** Clinical features of MRI observed in children (6 mo to < 5 years ) with CFS MCA, middle cerebral artery; ADEM, Acute disseminated encephalomyelitis; CSF, Cerebrospinal fluid; MRI, Magnetic resonance imaging

Features of MRI	N (%)
Normal Study	25 (69.44%)
Acute infarction of right MCA territory	2 (5.56%)
Bilateral symmetrical volume loss in the hippocampus, T2 flair hyperintensity in the bilateral frontoparietal region with cerebral atrophy and prominence in cerebral follae cortical and subcortical tubers in the bilateral frontoparietal region (Tuberous Sclerosis)	1 (2.78%)
Diffusion restriction in the right frontoparietal region	1 (2.78%)
Early Leukodystrophy changes	1 (2.78%)
Frontotemporal atrophy	1 (2.78%)
Gyri form diffusion restriction bilateral cerebral hemisphere most marked in the parietooccipital region	1 (2.78%)
Intra axial signal altered multifocal lesion involving bilateral cerebrum-ADEM	1 (2.78%)
Marked thickening of cortex and reduction in the sulcus, prominence of subarachnoid space, prominence of ventricles (Lissencephaly pachygyria spectrum)	1 (2.78%)
Subacute infarction of the right frontal lobe	1 (2.78%)
Total number of cases	36

In the next step of the analysis, we critically examined the influence of MRI findings on a few biological parameters in study children and tried to establish a correlation among them. The parameters were gender, age distribution, types of seizures, the pattern of seizure multiplicity, and postictal deficit. We found that out of 25 male patients, six (24%) patients had positive MRI findings, and from 11 female patients, five (45.45%) showed positive MRI findings. We did not find any significant difference correlation between MRI manifestations based on gender. Further analysis suggested that the incidence of positive MRI findings in patients from six months to 2.5 years of age is five (27.78%) and in cases aged 2.5 years to five years is six (33.33%).

In this study, out of a total of 31 GTCS cases, six (19%) had positive MRI findings, and out of a total of five focal convulsions cases, all (100%) had positive MRI findings (p=0.001) (Table 3). Besides, out of five focal convulsions, one (20%) had a single focal convulsion with positive MRI findings and four (80%) had multiple focal convulsions with positive MRI findings (p = 0.002).

**Table 3:** Association of seizure types in positive MRI findings among CFS cases GTCS, generalized tonic-clonic seizure; MRI, magnetic resonance imaging; CFS, cerebrospinal fluid

Type Seizure	MRI Finding			
	Absent	Present	Total	p-value
Focal	0 (0%)	5 (100%)	5 (100%)	0.001
GTCS	25 (80.65%)	6 (19.35%)	31 (100%)	Fisher exact
Total	25 (69.44%)	11 (30.56%)	36 (100%)	

In this study, out of 14 single episodes of seizure, five (35.71%) had positive MRI findings, and among 22 multiple episodes of seizure, six (27.27%) had positive MRI findings. In our study, among six cases with the postictal deficit, four (66.67%) had positive MRI findings, and among 30 without any postictal deficit, seven (23.33%) had positive MRI findings and there was no significant difference.

## Discussion

This study highlighted the association of MRI brain changes in CFS and found CNS lesions in 30.56% of patients. Earlier data suggested that MRI abnormalities were observed in 14.8% of children with complex febrile seizures [11]. The present study clearly indicates a higher number of cases of CFS with MRI brain changes in the form of cortical focal hyperintensity (CFH) (42.1%). This might be due to better delineation of the brain with the latest MRI machines as compared to the previous study in 2008 [11]. Also, MRI brain lesions are increasing with the progression of age.

The majority of the children with CFS are within three years of age. Similar findings are noted in a group of 33 patients with CFS (mean age, 17.8 months) [12]. The most common type of presentation of CFS in this study group is GTCS (86.11%). The number of multiple seizures among the group is 61.11%. A similar presentation by another study was noted [13].

Patients presenting with complex febrile seizure (CFS) can demonstrate a wide range of structural abnormalities of the brain that MRI can reliably identify and localize, thus enabling the execution of the plan for further management. In this study, 68.4% of cases of MRI brain abnormalities are cortical focal hyperintensity and sub-cortical focal hyperintensity. Similarly, subcortical focal hyperintensity, abnormal white matter signals, and focal cortical dysplasia were the most common CNS finding in cases of CFS as described by Hesdorffer and colleagues [11]. These results highlight the potential for a pre-existing lesion that predisposes one to long-lasting seizures. A recent review of febrile seizure suggests that abnormalities in the brain may decrease the threshold of seizure in febrile children, putting one at risk for CFS development [14]. As most of the cases of CFS were managed clinically, if MRI is mandated in these children with CFS, it may add value for appropriate management.

In this study, 19% of GTCS cases have positive MRI findings compared to all five (100%) focal convulsions cases that had positive MRI findings (p=0.001). These findings suggest that brain abnormalities, which are common in focal convulsions cases, decrease seizure threshold in children, and they are at risk for CFS development [15,16]. It is found that those cases that had multiple focal convulsions are more likely to have abnormal MRI findings ( p = 0.002). Other studies have shown similar findings [11,17,18]. Further, in cases with the postictal deficit, 66.67% had abnormal MRI findings. This is an important finding that will help the clinician with appropriate treatment early. So clinicians must be cautious of the organic pathology in the central nervous system in children with postictal states with CFS and anticonvulsants to be started accordingly.

We found the mean duration of hospitalization in subjects with MRI findings to be 12.6+2.8 days. A similar study in India on children admitted for epilepsy described a hospital stay of seven days (range of 4 to 20 days) [19]. However, another study from India showed the duration of hospital stay was three to seven days in 73% of CFS cases, and the mean duration of hospital stay for SFS and CFS was 1.29±0.576 days [20]. The longer duration of stay in patients with positive MRI is due to the longer course of illness, multiplicity of seizures, and time to stabilize the patients before taking neuroimaging. Also, many patients (68%) in the positive MRI findings group required pediatric intensive care unit admission, which increased the duration of hospital stay.

Our study has multiple aspects that provide a unique strength to the manuscript; these include MRI imaging being done within 72 hours of the seizure episode, the collection of detailed data, and the minimization of bias by taking the opinions of two radiologists. Further, in this study, we examined focal seizure (FS), a common childhood condition, and no previous radiological evaluation was done in a systematic manner. The study looked at the brain structure in the first FS and discovered that MRI abnormalities are linked to focal and prolonged febrile seizures. Compared to children with simple febrile convulsions, individuals with complicated febrile convulsions show an increased chance of developing epilepsy. However, no follow-up investigation for cases has been performed in this study.

## Conclusions

The current study, performed on the East Indian population, showed cortical focal hyperintensity to be the most common abnormality in MRI brains in cases of complex febrile seizure followed by the onset of subcortical focal hyperintensity. Significant neuroimaging findings were noted among a few CFS patients even in the absence of other signs or symptoms. Patients with CFS only showing features of focal seizure or prolonged seizure for 24 hours appear to be at particularly higher risk for neuroimaging findings.
